# Lung immune signatures define two groups of end-stage IPF patients

**DOI:** 10.1186/s12931-023-02546-8

**Published:** 2023-09-28

**Authors:** Tamara Cruz, Núria Mendoza, Sandra Casas-Recasens, Guillaume Noell, Fernanda Hernandez-Gonzalez, Alejandro Frino-Garcia, Xavi Alsina-Restoy, María Molina, Mauricio Rojas, Alvar Agustí, Jacobo Sellares, Rosa Faner

**Affiliations:** 1 Centro Investigación Biomédica en Red Enfermedades Respiratorias (CIBERES), Barcelona, Spain; 2 Fundació Clínic Per a La Recerca Biomèdica - IDIBAPS (FCRB-IDIBAPS), C/Casanova 143, Cellex, P2A, 08036 Barcelona, Spain; 3 University of Barcelona, Barcelona, Spain; 4 Department of Pulmonology, Hospital Clinic, University of Barcelona, Barcelona, Spain; 5 Interstitial Lung Disease Unit, Respiratory Department, University Hospital of Bellvitge, IDIBELL, Hospitalet de Llobregat (Barcelona), CIBERES, Barcelona, Spain; 6 Department of Internal Medicine, The Ohio State University Wexner Medical Center, Columbus, OH USA; 7 Biomedicine Department, University of Barcelona, Barcelona, Spain

**Keywords:** Immune-signatures, Transcriptome, Idiopathic pulmonary fibrosis, Flow cytometry

## Abstract

**Background:**

The role of the immune system in the pathobiology of Idiopathic Pulmonary Fibrosis (IPF) is controversial.

**Methods:**

To investigate it, we calculated immune signatures with Gene Set Variation Analysis (GSVA) and applied them to the lung transcriptome followed by unbiased cluster analysis of GSVA immune-enrichment scores, in 109 IPF patients from the Lung Tissue Research Consortium (LTRC). Results were validated experimentally using cell-based methods (flow cytometry) in lung tissue of IPF patients from the University of Pittsburgh (n = 26). Finally, differential gene expression and hypergeometric test were used to explore non-immune differences between clusters.

**Results:**

We identified two clusters (C#1 and C#2) of IPF patients of similar size in the LTRC dataset. C#1 included 58 patients (53%) with enrichment in GSVA immune signatures, particularly cytotoxic and memory T cells signatures, whereas C#2 included 51 patients (47%) with an overall lower expression of GSVA immune signatures (results were validated by flow cytometry with similar unbiased clustering generation). Differential gene expression between clusters identified differences in cilium, epithelial and secretory cell genes, all of them showing an inverse correlation with the immune response signatures. Notably, both clusters showed distinct features despite clinical similarities.

**Conclusions:**

In end-stage IPF lung tissue, we identified two clusters of patients with very different levels of immune signatures and gene expression but with similar clinical characteristics. Weather these immune clusters differentiate diverse disease trajectories remains unexplored.

**Supplementary Information:**

The online version contains supplementary material available at 10.1186/s12931-023-02546-8.

## Introduction

Idiopathic Pulmonary Fibrosis (IPF) is an interstitial lung disease of unknown origin characterized by progressive lung fibrosis [1]. The pathogenesis of IPF is complex and still unclear. Previous studies of whole genome transcriptomics have described alterations in different molecular pathways in end-stage IPF lungs, including aberrant activation of epithelial cells that promote fibroblast to myofibroblast differentiation [2, 3], excessive production of extracellular matrix proteins, such as matrix metalloproteases (MMPs), collagen and fibronectin [4, 5], aberrant activation of lung developmental pathways [6, 7], mitochondrial abnormalities [8, 9] and oxidative stress [9, 10, and type II epithelial cells and fibroblasts senescence [2, 11, 12]. The combination of all these pathogenic mechanisms leads to a highly heterogeneous disease, in which the identification of disease endotypes is an important unmet clinical need to move toward precision treatment [13].

In this setting, the role of the immune system is unclear. Some studies have proposed a role of immune pathways such as CD3 + and CD20 + lymphocytes in the development of fibrosis [14, 15] through the promotion of epithelial to mesenchymal transition (EMT) [4, 7, 15–17]. Further, the progression of IPF and the occurrence of exacerbations was associated with B cell responses [18, 19] through their capacity to modify the pro or anti-fibrotic lung micro-environment, thus influencing fibroblasts activity [20]. However, other findings challenge the role of the immune response in IPF [21]. First, clinical trials with immune-suppressive agents showed increased mortality and fibrosis in treated patients [22]. Second, the expression of markers of lung T lymphocytes exhaustion (such as PD-1, ICOS and CD28) is associated with enhanced TGF-β production and poor survival in IPF [23, 24]. Finally, the proportion of NK cells with impaired activity is reduced in IPF lungs [25] and their functionality is profoundly compromised by the lung microenvironment [26].

We therefore hypothesized that it is likely to be significant immune-related molecular heterogeneity in patients with IPF. To test this hypothesis, we used gene set variation analysis (GSVA) in lung tissue samples of patients with IPF, instead of previous studies using conventional analysis of single-gene expression. GSVA is a statistical technique that enables the discovery of inflammatory and leukocyte lineage gene signatures by comparing combined enrichment scores (ESs) of established and predefined gene sets, especially in heterogeneous samples [27, 28]. Specifically: (1) we first applied GSVA to lung transcriptomic data of 109 severe IPF patients (explanted lungs) available at the Lung Tissue Research Consortium (LTRC) to estimate the proportion of immune cells in their lungs; (2) we then used unbiased cluster analysis to identify distinct groups of IPF patients with overall distinct level of immune signatures; and, finally, (3) we explored differential gene expression between observed clusters, both for newly identified signatures as well as for previously stablished IPF related pathways.

## Methods

### Availability of data and materials

The datasets supporting the conclusions of this article are available in the NIH public repository Lung Tissue Research Consortium (LTRC), https://www.nhlbi.nih.gov/science/lung-tissue-research-consortium-ltrc. Tables with the full results of the analysis performed to support the conclusions are available in the online supplement.

### Study design, patients and ethics

Transcriptomic data of IPF explanted lungs (n = 109) was obtained from the LTRC following established procedures. Experimental validation using cell-based (not mRNA) methods (flow cytometry) was performed in lung tissue samples of IPF patients undergoing bilateral lung transplant at the University of Pittsburgh (USA). The Institutional Review Board and the Committee for Oversight of Research and Clinical Training Involved Decedents of the University of Pittsburgh, approved the study and the sample transfer respectively. In all cases, a signed informed consent form was collected before organ procurement.

### Clinical characterization of IPF patients

Available clinical data in LTRC include age, sex, body mass index, Forced Expiratory Volum (FEV1), Forced Volum Capacity (FVC), carbon monoxide diffusing capacity (DLCO), quantify Computed Tomography (CT) of the thorax by an adapted version of the CALIPER software and daily activity and health questionaries. All procedures were realized following LTRC protocols, the diagnosis of IPF was performed by a specialist evaluating the medical record, CT scan report and the post-transplant pathology report.

### GSVA, immune-signatures enrichment and unbiased cluster analysis

We analyzed the transcriptomic data set GSE47460 from the LTRC [29]. This data set was split in two, GPL14550 was used as a discovery data set (D#1, n = 109) whereas GPL6480 was used for validation (D#2, n = 34). For the current analysis we used the normalized matrix downloaded from GEO, selecting only patients with a diagnosis of IPF. Gene set variation analysis (GSVA) was used to determine patient‐wise enrichment scores (ES) that indicate the relative collective expression of genes within the gene signatures for patients relative to the rest of the cohort of patients in a given transcriptomic dataset [30]. Sets of the immune signatures used were based on available gene expression publications (n = 31, Additional file 2: Table S1) [27, 31]. Unbiased clustering of the GSVA immune signatures were identified using the dendextend R package in R [32]. To maximize the differences in the GSVA scores, the number of clusters was set at 2, the distance metric was calculated with the minkowski method and the hierarchical clustering method was ward. D2 [32].

Differential gene expression between clusters was investigated using limma [33]. To build the correlation network with the clinical parameters and to further understand the relationship between the immune and epithelial cells in these patients, the gene sets included in our GSVA analysis were extended, while preserving the already obtained immune-based unbiased clustering, to include epithelial lineage cell signatures (skipping genes already included in the immune cell signatures) (Additional file 2: Table S2).

### Experimental validation of LTRC results in fresh lung tissue samples by flow cytometry

To validate results from the GSVA immune enrichment in the LTRC, we used flow cytometry, a non-mRNA related method. Fresh lung tissue samples of IPF patients undergoing bilateral lung transplant at the University of Pittsburgh (USA) were washed with PBS and enzymatically digested as previously described [34]. Lung homogenates included multiple areas of the same lung lobe, ensuring the representability of the sample to address patient’s heterogeneity. Lung tissue homogenates (10^6^ cells) were then stained 5 min with the viability staining (Fixable viability-Alexa600, BD, USA) and 30 min at 4ºC in the dark with the following conjugated monoclonal antibodies CD3-PECy5.5, CD45-Alexa700, CD16-BV412, CD56-FITC, CD8-V500, CD4-APC-Cy7, CD19-BV650 (BD, USA) and CD14-PE (BioLegend, USA). A minimum of 5 × 10^5^ cells per sample were acquired in a FACS LSRII (BD Biosciences, USA), and data was analyzed using FlowJo v10 (FlowJo LLC, USA). Immune cell populations were determined using the gating strategy depicted in Additional file 1: Fig. S1.

### Biologic pathway analysis

To evaluate the enrichment of biological signatures in the observed clusters, gene ontology (GO) enrichment and hypergeometric tests were used [35]. The gene signatures for the hypergeometric test were selected from previously published sc-RNAseq studies: epithelial cells signatures [36–39] and fibroblast related signatures [37–41]; or from the Gene Ontology (GO) extracellular matrix (GO:0031012), oxidative stress (GO:0000302), mitochondrial transport (GO:0006839), mitochondrial respiratory chain (GO:0005746) and response to stress (GO:0006950). Additional file 2: Table S3 shows the complete list of gene signatures investigated here.

### Statistical analysis

Quantitative and qualitative data is presented as mean, or n and proportion, respectively. Results were compared using the ANOVA or Fisher tests, as appropriated. Differences in the distribution of the GSVA calculated signatures between clusters were assessed with the ANOVA test too. Correlations between immune cell signatures and clinical features were assessed using the Spearman correlation test, which was considered statistically significant if its r value was >|0.5| and the p value < 0.05. To explore correlations between biological and clinical features, we used network analysis, where each node was the variable of interest, its size was proportional to its mean value in each cluster, and links (edges) represent the Spearman Rho between linked variables, with results being plotted using Cytoscape [42]. All statistics were computed with R 4.2.2, using custom scripts.

## Results

### Cluster analysis of enriched immune-signatures in the LTRC

**Fig. 1:**
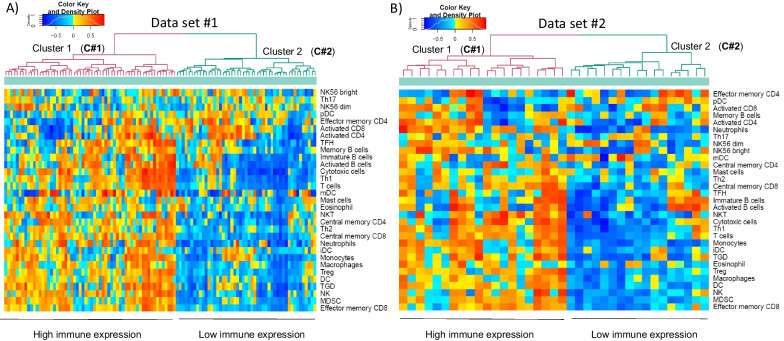
Unbiased clustering of obtained GSVA-immune enrichment scores, in the IPF LTRC samples. **A** Data-set 1 and **B** Data-set 2. The density color keys at the top left of each figure define the scoring for each gene signature ranging from − 1 in blue to 1 in red

The main demographic and clinical characteristics of IPF patients included in D#1 and D#2 were similar (Additional file 2: Table S4). Briefly, the studied population presented the clinical characteristics of end-stage IPF disease, a severe impairment of the DLCO and FVC, and fibrotic features in the CT scan, presence of honeycombing, ground grass opacity, reticular densities and vessels. As shown in Fig. 1, in both data-sets (panels A and B) k-means unbiased clustering of GSVA enriched immune signatures identified two clusters of IPF patients (C#1 and C#2) with different levels of immune expression. Additional file 2: Table S5 shows the mean ES in each cluster and the p-value for the comparison of both clusters. C#1 had a higher ES than C#2 in all analyzed immune signatures except for three of them where no significant differences were observed between clusters. The biggest differences were found in cytotoxic cells (both adaptive CD8 + T cells and innate NK lymphocytes) and memory T cells.

Table 1 compares the main clinical differences between IPF patients included in the two clusters (C#1 and C#2) identified in D#1. Briefly, C#1 (high immune expression) included slightly younger individuals, with more symptoms, and less low attenuation area by CT scan. These differences were reproduced in the two clusters determined in D#2 (Fig. 1B and Additional file 2: Table S6).

**Table 1:** Main clinical characteristics of the two IPF GSVA clusters in D#1. Statistically significant results are highlighted in bold

	Cluster 1			Cluster 2			p-value
	n	mean	SD	n	mean	SD	
Age	54	63.67	8.73	46	66.91	7.24	**0.048**
Weight (kg)	55	88.36	17.31	47	91.73	18.05	0.338
BMI	55	30.48	4.97	47	30.67	5.82	0.852
Smoking (pack/year)	39	28.45	24.3	27	23.02	18.25	0.329
Quit smoking (years)	39	21.11	13.47	27	24.25	13.92	0.361
GAP score (1–7)	60	4.64	1.23	49	4.94	1.16	0.207
Lung function test							
Predicted DLCO	55	29.37	5.69	47	30.35	4.9	0.357
Predicted FEV1	55	69.56	16.54	47	73.77	18.74	0.232
Predicted FVC	55	63.15	14.87	47	66.57	18.43	0.301
FEV1/FVC	55	0.83	0.06	47	0.83	0.06	0.973
TAC							
Total Segmented Volume with density less than -950 HU (cm^2^)	29	155.27	140.98	21	244.15	164.3	**0.044**
Lower Attenuation areas (cm^2^)	29	4.38	3.42	21	6.08	3.22	0.080
Ground Glass Opacity (cm^2^)	29	17.15	14.38	21	11.22	11.11	0.119
Honeycombing (cm^2^)	29	1.2	1.95	21	1.27	1.24	0.893
Normal (cm^2^)	29	52.86	13.69	21	46.99	16.22	0.169
Reticular densities (cm^2^)	29	4.14	3.03	21	4.47	3.56	0.719
Vessels (cm^2^)	29	5.62	2	21	5.14	1.88	0.398
Categorical variables							
Short of breath when talk, n (%)	60	34 (56.7)		49	16 (32.6)		**0.023**
Cough disturbs sleep, n (%)	60	37 (45)		49	10 (20.4)		**0.014**
Long time to wash or dress, n (%)	60	22 (36.7)		49	6 (2.2)		**0.007**
Chronic bronchitis, n (%)	60	7 (12.1)		49	0 (0.0)		**0.044**

A sensitivity analysis, done using only the data of upper lobes or lower lobes showed that there were no differences in the immune signatures enrichment in upper *vs*. lower lung lobes (Additional file 1: Figure S2 and Additional file 2: Table S7, S8). Likewise, the direct comparison between different lobes did not identify differences in the immune-signatures ES, although we found the expected differences in CT scan parameters with increased extend of the fibrosis related parameters in the lower lobes (Additional file 2: Table S9).

### Validation of results by flow cytometry in fresh lung tissue

**Fig. 2:**
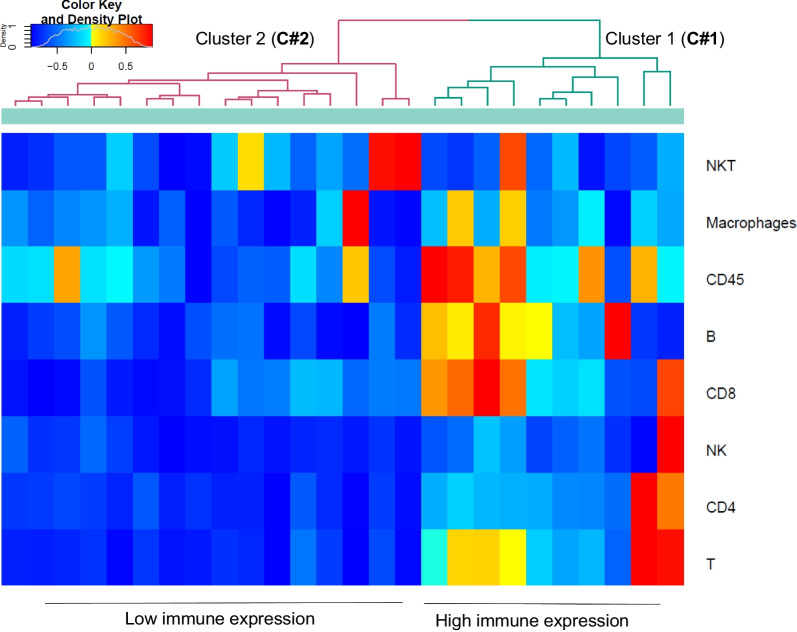
Unbiased clustering of the flow cytometry data generated for the validation. Flow cytometry determination of the main lung immune populations followed by unbiased clustering showed the presence of two clusters of IPF patients based on their level of immune infiltrate in fresh lung samples. The density color key at the top left define the scoring for each gene signature ranging from (− 1) in blue to (1) in red

To validate the above discussed results, we used flow cytometry in fresh lung tissue samples harvested from IPF explanted lungs (Additional file 2: Table S10). Further, to exclude the possibility that the two clusters identified above may actually correspond to pathology heterogeneity within the sampled lung lobe rather than differences between patients, for flow cytometry measurements we used lung homogenates from multiple areas to ensure proper representation of the whole pulmonary lobe. By doing so, unbiased clustering of flow cytometry data confirmed the existence of two clusters of patients that differed in the proportion of T-cells, CD4, CD8, B-cells, NK cells, NKT-like cells and macrophages (Fig. 2A).

### Biological pathways

To gain insight into the biological process altered in the two clusters of IPF patients identified from the immune signatures enrichment in the LTRC, we investigated differentially express genes (DEG) in C#1 and C#2. Using an adjusted p value < 0.05 and log Fold Change (LgFC) >|0.65| we found 777 DEG; 153 (19.7%) of them were upregulated in C#1 and 624 (80.3%) in C#2 (Additional file 2: Table S11).

**Fig. 3:**
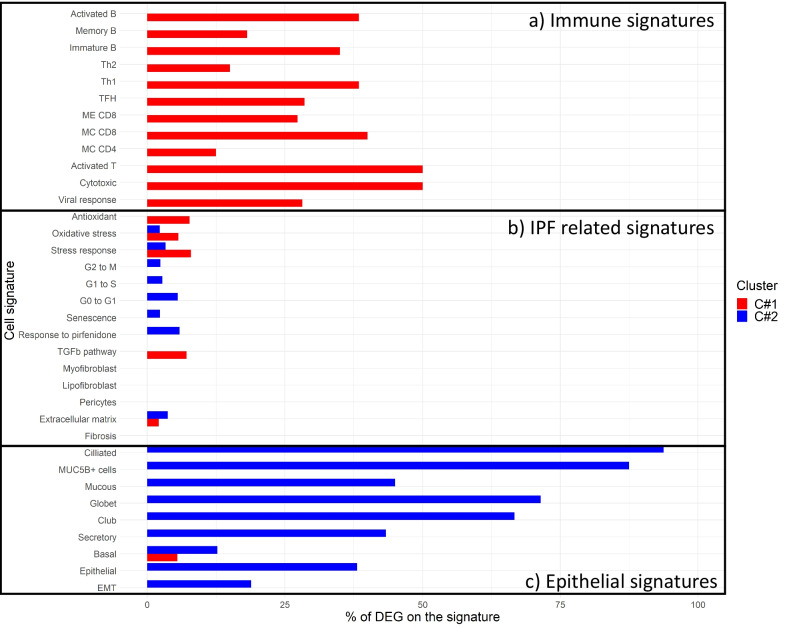
Hypergeometric test of the percentage of cluster differentially express genes in the studied biological gene signatures. **A** Immune signatures, **B** IPF related signatures and **C** epithelial signatures

Additional file 2: Table S12 lists the biological ontologies associated with these DEG. Of note, C#1 showed activation of immune response ontologies whereas C#2 included ontologies related to ciliary function. To contrast these results with pathways previously reported in IPF, we performed a hypergeometric test on DEG with specific IPF related signatures, including epithelial lineage, cell cycle, senescence, extracellular matrix, myofibroblast activation, response to pirfenidone treatment, oxidative stress, endoplasmic reticulum stress, mitochondrial related genes and immune lineage (Additional file 2: Table S3). We observed that C#1 showed increased viral response and immune infiltrate gene signature, thus supporting GSVA unbiased clustering results. By contrast, C#2 was characterized by altered epithelial cell lineage (Fig. 3 and Table 2), particularly upregulation of genes related to EMT, secretory and ciliated cells. Interestingly, there were no differences between C#1 and C#2 in fibrosis associated gene signatures (Fig. 3 and Table 2).

**Table 2:** Hypergeometric test comparing the percentage of differentially express genes between clusters that belong to the following specific signatures. Statistically significant results are highlighted in bold

	Cluster 1			Cluster 2		
	OR	Matched genes (%)	p-value	OR	Matched genes (%)	p-value
Immune signatures						
Activated T cells	59.30	50.00	**1.62E−03**	0	0	1
Activated B cells	37.55	38.46	**1.43E−06**	0	0	1
Activated CD4	0	0	1	0	0	1
Activated CD8	0	0	1	0	0	1
Central memory CD4	8.48	12.50	**0.03**	0	0	1
Central memory CD8	40.20	40.00	**8.92E−08**	0	0	1
Cytotoxic cells	59.78	50.00	**5.00E−06**	0	0	1
Effector memory CD4	0	0	1	1.85	8.33	0.44
Effector memory CD8	22.88	27.27	**2.34E−09**	0	0	1
Immature B cells	32.60	35.00	**2.12E−08**	0	0	1
Memory B cells	13.20	18.18	**0.01**	0	0	1
NK cells	4.55	7.14	0.21	0	0	1
NK CD56 bright	0	0	1	0	0	1
NK CD56 dim	0	0	1	0	0	1
NKT cells	9.86	14.29	0.11	0	0	1
T follicular helper	23.75	28.57	**5.50E−03**	0	0	1
TGD	5.76	8.82	**0.02**	0	0	1
Th1	38.30	38.46	**5.82E−12**	0	0	1
Th17	0	0	1	1.07	5.00	0.62
Th2	11	15	0	0.00	0.00	1.00
Viral response	24.15	28.20	**2.46E−11**	0	0	1
Epithelial signatures						
EMT	0	0	1	4.79	18.87	**1.49E−04**
Epithelial	0	0	1	12.65	38.10	**2.63E−06**
Basal cells	3.43	5.45	0.06	2.99	12.73	**0.01**
Activated basals	5.38	8.33	0.18	1.85	8.33	0.44
Aberrant basaloid	0	0	1	0	0	1
Primed basals	6.58	10.00	0.15	8.75	30.00	**0.01**
Proliferative basals	0	0	1.00	0	0	1
Multipotent basal	3.29	5.26	0.27	3.83	15.79	0.06
Secretory cells	0	0	1	15.82	43.33	**2.68E−10**
Club cells	0	0	1	40.89	66.67	**6.64E−05**
Globet cells	0	0	1	51.53	71.43	**4.03E−11**
Mucous cells	0	0	1	16.85	45.00	**1.09E−07**
Serous cells	0	0	1	7.98	28.00	**1.10E−04**
MUC5Bpos	0	0	1	143.70	87.50	**3.69E−09**
Cilliated cells	0	0	1	4.26	17.24	**0.01**
Cilliated cells type 1	0	0	1	82.57	80.00	**4.07E−14**
Cilliated cells type 2	0	0	1	102.45	83.33	**1.28E−06**
Differentiated cilliated	0	0	1	309.85	93.75	**1.52E−19**
IPF cilium associated signatures [11]						
Pattern A	0	0	1	2139.52	98.92	**1.23E−123**
Pattern B	1.00	1.67	0.64	54.66	71.67	**3.64E−44**
Fibrosis associated signatures						
Fibrosis	0	0	1	0	0	1
Fibroblast activation	0	0	1	0	0	1
Lipofibroblast	0	0	1	0	0	1
Myofibroblast	0	0	1	0	0	1
Smooth muscle cells	0	0	1	0	0	1
Myofibroblast	0	0	1	0	0	1
Pericytes	0	0	1	0	0	1
Extracellular matrix	1.2	2.12	0.36	0.47	3.72	0.99
Matrix features	0	0	1	0	0	1
Response to pirfenidone	0	0	1	1.27	5.88	0.56
TGFb signaling	4.23	7.14	0.22	0	0	1
Senescence	0	0	1	0.48	2.33	0.87
G0 to early G1	0	0	1	1.20	5.56	0.58
G1 to S cycle	0	0	1	0.57	2.76	0.91
G2 to M cycle	0	0	1	0.49	2.37	0.96
Mitochondrial transport	1.21	2.17	0.57	0.28	2.17	0.96
Mitochondrial respiratory chain	0	0	1	0	0	1
Response to stress	5.25	7.94	**4.50E−08**	0.43	3.34	0.99
Oxidative stress	3.37	5.68	**0.02**	0.29	2.27	0.99
Pro-oxidant	5.82	9.52	**0.05**	0	0	1
Oxidative response	5.5	9.09	0.18	0	0	1
Antioxidant	4.61	7.69	0.07	0	0	1

### Network analysis

Finally, to further understand the relationship between the immune and epithelial cells in these IPF patients, the GSVA analysis was extended by adding the epithelial lineage cell signatures (Additional file 2: Table S2) and correlation networks were built considering the transcriptomic immune and epithelial signatures enrichment and the clinical characteristics of the patients. Of note, to analyze the relationship between CT findings and cell signatures we used only CT measures in the profiled pulmonary lobe. Additional file 2: Figure S3 shows a first neighbor correlation network of the clinical parameters and epithelial signatures in C#1 and C#2. In both clusters we observed a negative correlation between epithelial and immune cells (dashed edges (links)). Specifically, epithelial, ciliated, and secretory cell signatures were negatively correlated with central memory CD8 + T cells, Th2 T cells and immature B cells. Interestingly, only in C#2 we identified a correlation between the transcriptomic signatures and disease severity: a positive correlation with fibrosis associated CT parameters and a negative correlation with the FVC value.

## Discussion

The main and novel observation of this study are that, by using unbiased cluster analysis of lung immune signatures in a large cohort of patients with IPF (n = 109), we identified two clusters (C#1 and C#2) of similar size with different immune-related characteristics and differentially expressed genes: C#1 (n = 55, 53%) was characterized by a higher expression of immune signatures, particularly cytotoxic and memory T cells, whereas C#2 (n = 49, 47%) was characterized by an upregulated expression of cilium associated genes, epithelial and secretory cells (structural cell cluster). Interestingly, though, the clinical presentation of these two clusters was remarkably similar, indicating that at the end-stage of the disease the identified molecular heterogeneity does not translate directly into a different clinical phenotype. However, further research is need to understand whether these clusters are already present in earlier phases of the disease and/or associated with the disease progression.

## Previous studies

A few previous studies used transcriptomic data to identify clusters of IPF patients. Using lung transcriptomics, Yang et al*.* identified a cilium associated subtype and a fatty acid metabolism one [43], but the expression of immune related genes or the associated cell types was not reported. Using blood transcriptomics, Kraven et al. described three clusters of IPF patients, one of them enriched in immune response genes [44]. Additionally, Herazo-Maya JD et al. identified a 52 gene signature on PBMCs that stratified patients with different disease outcomes [45, 46], and an increase of peripheral blood monocytes has been associated with poor prognosis [47]. Finally, De Sadeleer et al. used transcriptomic results of bronchoalveolar lavage fluid analysis identified 6 clusters in IPF patients, one of them again enriched in immune signatures [48]. Collectively, these studies support our observation of immune heterogeneity in IPF. To our knowledge, however, no previous study has used unbiased cluster analysis of IPF lung immune signatures enrichment. Importantly, results were validated experimentally in independent lung tissue samples using non-mRNA related method (flow cytometry).

## Interpretation of novel findings

The application of this cutting-edge methodology to IPF lung tissue allowed us to identify two clusters of IPF patients (C#1 and C#2) with marked biological differences: while C#1 was an "*immune-cell*" cluster, particularly enriched in cytotoxic and memory T cells, C#2 was a "*structural cell*" cluster, with marked upregulation of cilium, epithelial and secretory cells genes. Because in the study mentioned above Yang et al*.* also identified a cilium associated IPF subtype using lung transcriptomics [11], we explored the degree of overlap between their results and our identified clusters. The hypergeometric test showed that our C#2 shared a 99% and 72% of their described genes, indicating that our unbiased clustering of immune signature enrichment generates a similar grouping of IPF patients than the more traditional transcriptomic hierarchical clustering.

From the clinical viewpoint, it is of note that these two very different biologic clusters of patients with IPF show remarkably similar clinical characteristics (Table 1). We think that this may likely be due to the fact that lungs were harvested at transplantation, this is at an end-stage course of the disease. It is possible that at an earlier stage, clinical differences may have been more evident or that these two clusters represent different disease trajectories, varying in either rate of progression, frequency of infections or exacerbations and/or the response to treatment. All these possibilities require and deserve future research. This is the main limitation of the study, the lack of longitudinal information to understand the disease evolution, progression and a record of infections and exacerbations that could have a direct impact in the lung immunological state.

## Conclusions

The use of unbiased clustering of the transcriptomic enrichment in immune signatures in lung tissue of patients with end-stage IPF identified two distinct clusters, an immune-cell one and a structural-cell one, with a negative correlation between the expression of immune and epithelial related signatures. These very different biological clusters are not related with clinical characteristics but whether they are present at an earlier stage and/or there is an association with disease phenotypes or progression should be further studied.

### Supplementary Information

**Additional file 1:** **Figure S1.** Flow cytometry gating strategy to identify the main immune populations in the lung**.** Cell debris is excluded and single cells are selected using FSC VS SSC. Live cells are selected using a cell viability marker and the hematopoietic cells are selected as CD45 + . Macrophages/monocytes are selected by gating the CD14 population. For lymphocyte determinations complex cells are excluded based on the SSC to reduce the lung autofluorescence. From this lymphocyte population, B lymphocytes are selected as CD19 + , NK cells are selected as CD3-CD56 + and T cells are CD3 + . CD4 + and CD8 + T lymphocytes are selected from the CD3 + population. **Figure S2**. GSVA unbiased clustering of the IPF samples dividing by the profiled lung lobe**. A** Upper lobe **B** Lower lobe. **Figure S3.** First neighbor correlation networks. **A** Cluster 1 **B** Cluster 2. Nodes represent the FC of the median between the 2 clusters. Lung clinical parameters are represented in octagons (CT scan are light green and pulmonary function test parameters are dark green), GSVA cell types in circles (lung cell types are turquois, cytotoxic cells are yellow, T cells are blue, B cells are pink and innate cells are light purple) and the biological pathways are denoted in rhombus. The width of the edges represents the correlation coefficient, negative correlations are marked with dotted lines and positive correlations are indicated with solid gray. R >|0.5| and p < 0.05.

**Additional file 2:** **Table S1.** Immune gene set signatures used in the GSVA pipeline to performed the unbiased clustering. **Table S2.** Extended gene set signatures with the epithelial and fibrosis associated signatures used in the GSVA to generate the correlation networks. **Table S3.** Hypergeometric test gene signatures for immune, epithelial and fibrosis related signatures. **Table S4.** Main clinical characteristics of D#1 y D#2. **Table S5.** Differences in immune cell signatures across clusters on D#1. **Table S6.** Differences in clinical characteristics and immune cell signatures across clusters on D#2. **Table S7.** Differences in clinical characteristics and immune cell signatures across clusters on D#1 filtering by upper lobes. **Table S8.** Differences in clinical characteristics and immune cell signatures across clusters on D#1 filtering by lower lobes. **Table S9.** Differences in clinical characteristics and immune cell signatures between upper and lower lobe. Results are expressed as median [95% coefficient interval] or mean (SD) as appropriate. **Table S10.** Main clinical characteristics of the validation cohort. **Table S11.** Statistically significant (adjusted p-value < 0.05) genes on the limma test of cluster 2 over cluster 1. **Table S12.** Gene ontologies enrichment of differentially express genes between the 2 clusters. On the left side the gene ontologies of the cluster 1 are represented (negative values of LgFC Cluster 2/Cluster1), on the right side the gene ontologies of the cluster 2 are represented (positive values of LgFC Cluster 2/Cluster1).

## Data Availability

The datasets supporting the conclusions of this article are available in the NIH public repository Lung Tissue Research Consortium (LTRC) 45. Tables with the full results of the analysis performed to support the conclusions are available in the online supplement.

## Abbreviations

IPF: Idiopathic Pulmonary Fibrosis
GSVA: Gene Set Variation Analysis
LTRC: Lung Tissue Research Consortium
C#: Cluster
NIH: National Institute of Health
mRNA: Messenger Ribonucleic Acid
FEV1: Force Expiratory Volume in 1 s
FVC: Force Vital Capacity
DLCO: Diffusing Capacity of Carbon Monoxide
CT: Computerized Tomography
ES: Enrichment Score
CD: Cluster of Differentiation
FACS: Fluorescence Activated Cell Sorting
sc-RNAseq: Single Cell RNA Sequencing
GO: Gene Ontology
ANNOVA: Analysis of Variance
NK: Natural Killer
D#: Dataset
NKT-like: Natural Killer T-cell like
DEG: Differentially Express Genes
LgFC: Logarithmic Fold Change
EMT: Epithelial Mesenchymal Transition
Th: T-cell Helper response
