# Supplementary figures and images for: Lung immune signatures define two groups of end-stage IPF patients
Source: Respir Res. 2023 Sep 28;24:236. doi: 10.1186/s12931-023-02546-8 (PMC10540496; doi:10.1186/s12931-023-02546-8)

## Slide 1
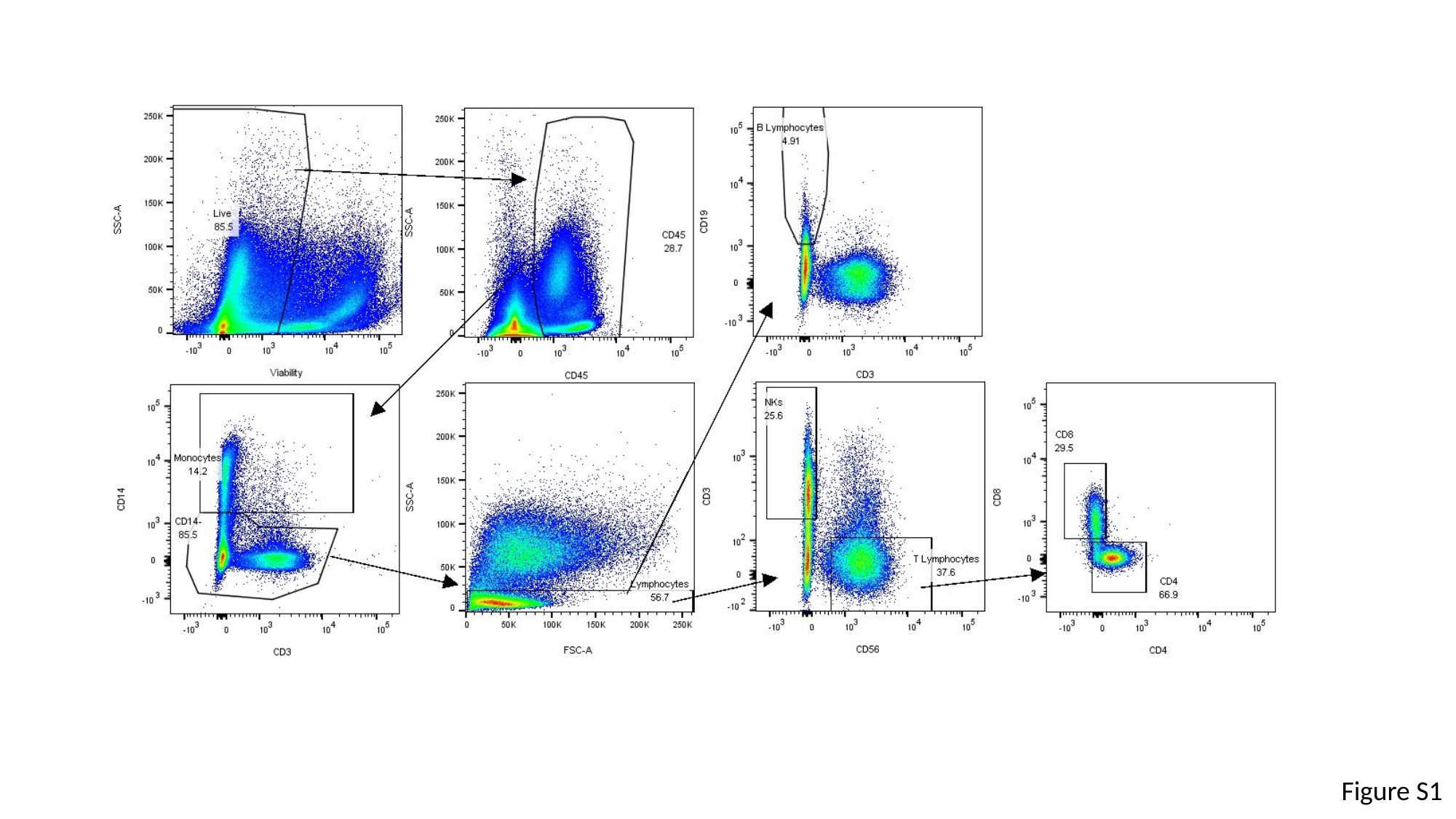

Figure S1

## Slide 2
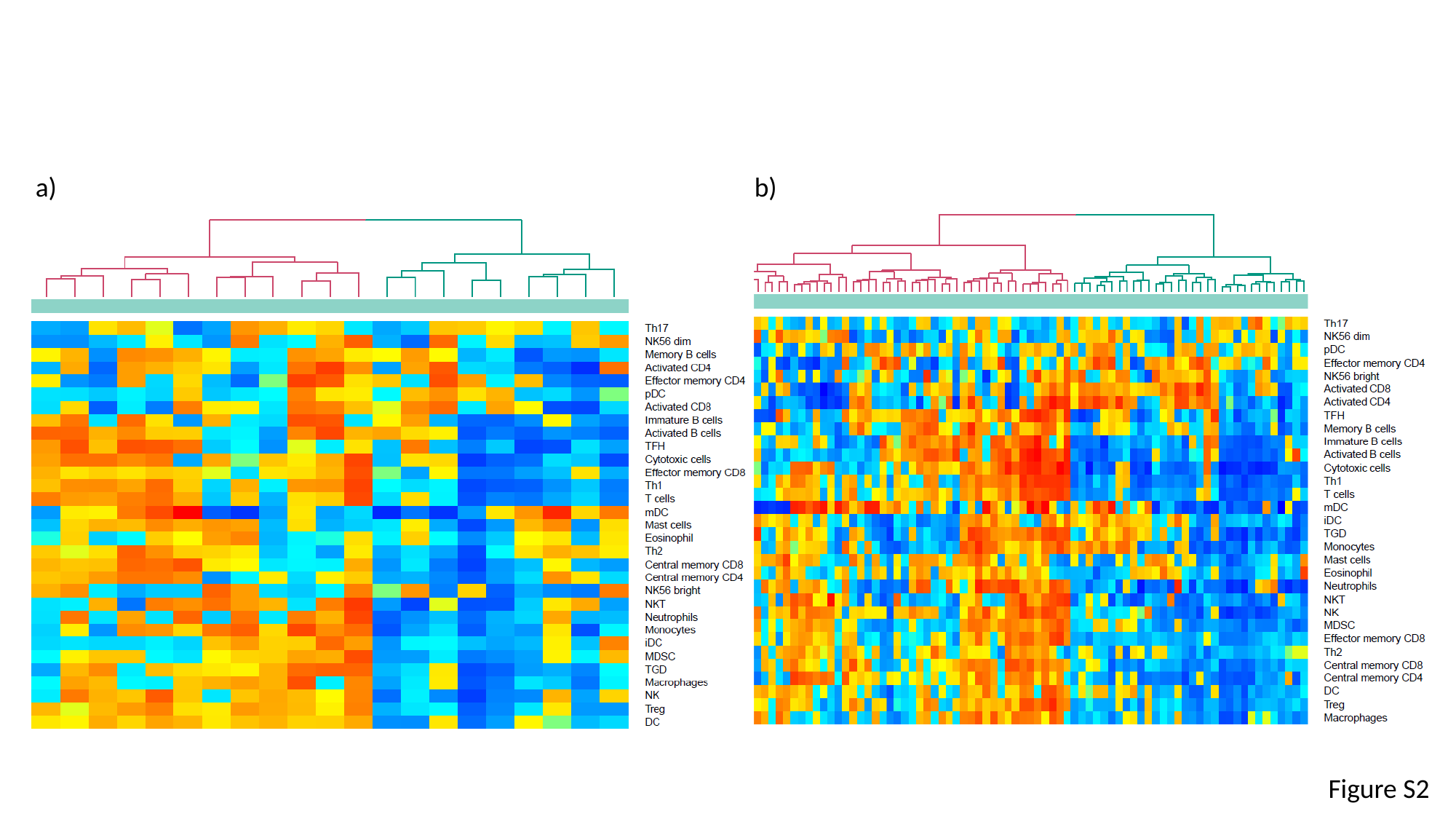

a)
b)
Figure S2

## Slide 3
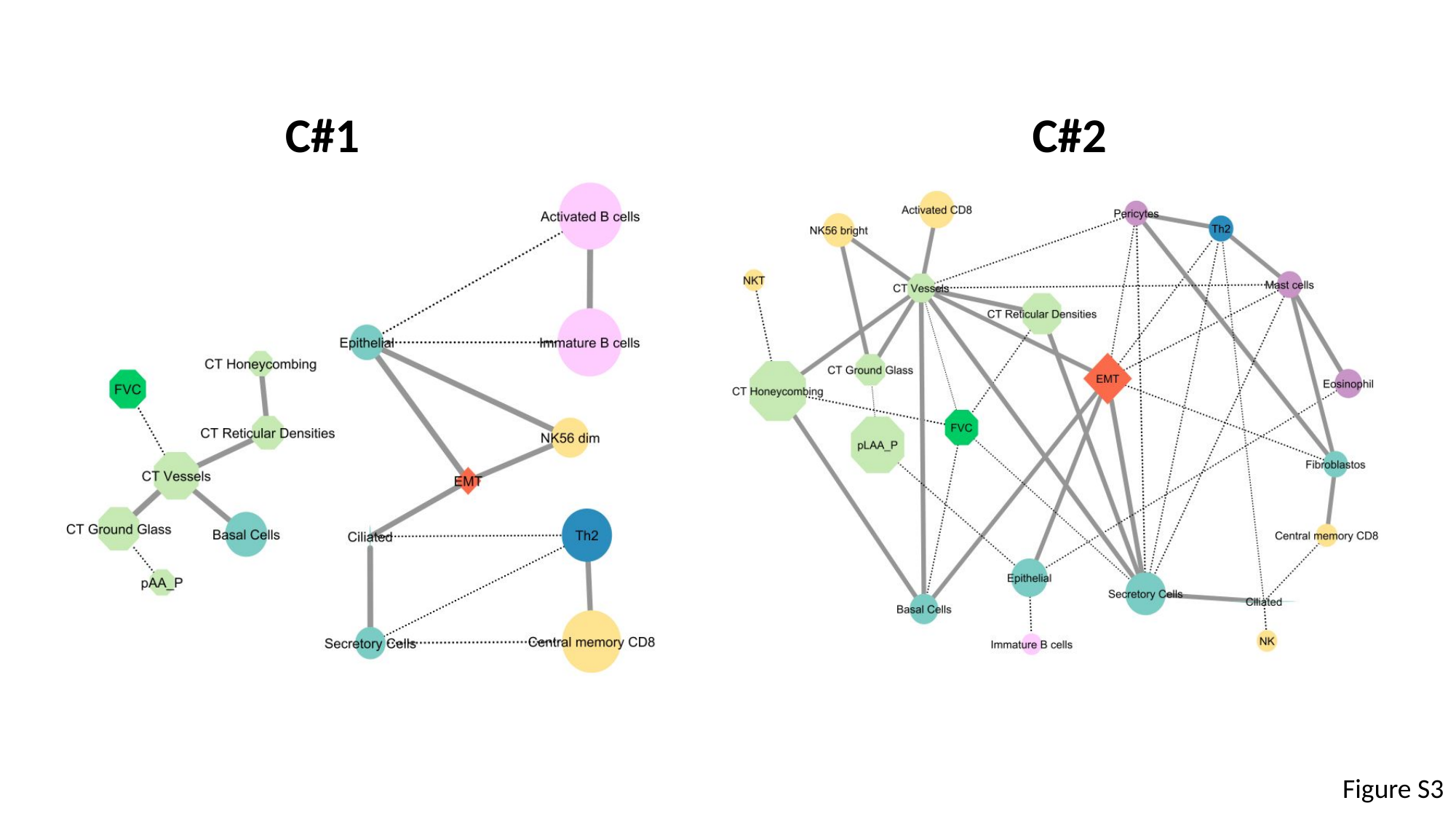

C#1
C#2
Figure S3

Supplement: Supplementary file 1 — Additional file 1: Figure S1. Flow cytometry gating strategy to identify the main immune populations in the lung. Cell debris is excluded and single cells are selected using FSC VS SSC. Live cells are selected using a cell viability marker and the hematopoietic cells are selected as CD45 + . Macrophages/monocytes are selected by gating the CD14 population. For lymphocyte determinations complex cells are excluded based on the SSC to reduce the lung autofluorescence. From this lymphocyte population, B lymphocytes are selected as CD19 + , NK cells are selected as CD3-CD56 + and T cells are CD3 + . CD4 + and CD8 + T lymphocytes are selected from the CD3 + population. Figure S2. GSVA unbiased clustering of the IPF samples dividing by the profiled lung lobe. A Upper lobe B Lower lobe. Figure S3. First neighbor correlation networks. A Cluster 1 B Cluster 2. Nodes represent the FC of the median between the 2 clusters. Lung clinical parameters are represented in octagons (CT scan are light green and pulmonary function test parameters are dark green), GSVA cell types in circles (lung cell types are turquois, cytotoxic cells are yellow, T cells are blue, B cells are pink and innate cells are light purple) and the biological pathways are denoted in rhombus. The width of the edges represents the correlation coefficient, negative correlations are marked with dotted lines and positive correlations are indicated with solid gray. R >|0.5| and p < 0.05. [file 12931_2023_2546_MOESM1_ESM.pptx]
